# Reciprocal Interactions of Mitochondria and the Neuroimmunoendocrine System in Neurodegenerative Disorders: An Important Role for Melatonin Regulation

**DOI:** 10.3389/fphys.2018.00199

**Published:** 2018-03-12

**Authors:** Victoria O. Polyakova, Igor M. Kvetnoy, George Anderson, Jessica Rosati, Gianluigi Mazzoccoli, Natalya S. Linkova

**Affiliations:** ^1^Department of Gynecology and Reproductology, Ott Institute of Obstetrics, Saint Petersburg, Russia; ^2^Department of Cell Biology and Pathology, Saint-Petersburg Institute of Bioregulation and Gerontology, Saint Petersburg, Russia; ^3^Department of Physiology and Department of Pathology, Saint Petersburg State University, Saint Petersburg, Russia; ^4^CRC Scotland and London Clinical Research, London, United Kingdom; ^5^Cell Reprogramming Unit, IRCCS “Casa Sollievo della Sofferenza”, San Giovanni Rotondo, Italy; ^6^Division of Internal Medicine and Chronobiology Unit, Department of Medical Sciences, IRCCS “Casa Sollievo della Sofferenza”, San Giovanni Rotondo, Italy; ^7^Peter the Great Saint Petersburg Polytechnic University, Saint Petersburg, Russia

**Keywords:** DNIES, mitochondrion, neuroimmunoendocrine, neurodegeneration, melatonin

## Abstract

Structural and functional alterations of mitochondria are intimately linked to a wide array of medical conditions. Many factors are involved in the regulation of mitochondrial function, including cytokines, chaperones, chemokines, neurosteroids, and ubiquitins. The role of diffusely located cells of the neuroendocrine system, including biogenic amines and peptide hormones, in the management of mitochondrial function, as well as the role of altered mitochondrial function in the regulation of these cells and system, is an area of intense investigation. The current article looks at the interactions among the cells of the neuronal-glia, immune and endocrine systems, namely the diffuse neuroimmunoendocrine system (DNIES), and how DNIES interacts with mitochondrial function. Whilst changes in DNIES can impact on mitochondrial function, local, and systemic alterations in mitochondrial function can alter the component systems of DNIES and their interactions. This has etiological, course, and treatment implications for a wide range of medical conditions, including neurodegenerative disorders. Available data on the role of melatonin in these interactions, at cellular and system levels, are reviewed, with directions for future research indicated.

## Introduction

Mitochondria are the sites of cellular energy production in eukaryotic cells and an important source of cellular second-messenger molecules, including hydrogen peroxide and other pro-oxidants. Such reactive oxygen intermediates are involved in many gene regulatory pathways. In metabolically active cells, mitochondria are the most abundant organelles, with 10–20% of total intracellular proteins present within this organelle (Suárez-Rivero et al., 2016).

Although mitochondria have their own DNA, encoding mitochondrial tRNA and several polypeptides, most mitochondrial proteins are imported from the cytoplasm. This import process faces the challenge of routing proteins to their correct sub-mitochondrial compartment, often involving their transport across two membranes. This challenge is met by the joint action of two distinct protein transport systems, in the outer and inner membranes.

Mitochondrial dysfunction is implicated in over 100 medical conditions. Structural and functional mitochondrial alterations are present in a wide variety of clinical disorders, often referred to as “*mitochondrial diseases*” (El-Hattab and Scaglia, 2016; Turnbull and Rustin, 2016). Genetic defects in the synthesis, transport and function of mitochondrial proteins are relevant to diverse clinical presentations, including in the central nervous system and liver, as well as skeletal and cardiac muscle.

The transport of mitochondrial proteins can be regulated by an array of compounds, including cytokines, chemokines, chaperones, ubiquitins, and neurosteroids (Gutiérrez and Simmen, 2017; Peña-Blanco et al., 2017). As such, alterations in mitochondrial function are intimately linked to local inter-cellular as well as intra-cellular signaling, with variations in mitochondrial output impacting on inter-cellular and intra-cellular processes. The roles of widely expressed biogenic amines, including dopamine, noradrenaline, adrenaline, histamine, and serotonin, as well as peptide hormones in the mechanisms underpinning mitochondrial dysfunction are at the cutting edge of research.

Alterations in mitochondrial function are also important to the activity of all biological systems, including neuronal-glia, immune, and endocrine systems. As such, mitochondrial functioning may be seen as in a complex two-way interaction with wider system functioning (Chow et al., 2017). This manuscript reviews the recent data regarding such interactions, highlighting the emerging body of data indicating a role for the melatonergic pathways in such processes, especially in the regulation of mitochondrial functioning.

## Development of the diffuse neuroimmunoendocrine system

In the 1960's, Pearse proposed that specialized, highly organized cell systems exist, with component cells able to produce peptide hormones and biogenic amines in different organs (Pearse, 1968, 1969). Subsequent experiments highlighted the common ability of these cells to absorb monoamine precursors (5-hydroxytryptophan and L-dihydroxyphenylalanine), producing biogenic amines in a process termed amine precursor uptake and decarboxylation (APUD) (Pearse, 1968, 1969; Neupert, 1997; Suárez-Rivero et al., 2016). The APUD series includes over 60 types of endocrine cells located at many sites, including in the intestine, brain and other glands, and organs (Bussolati, 2014).

Such a growing body of data challenged traditional conceptualizations of a hierarchical organization of the nervous and endocrine systems. This was replaced by a framework emphasizing the coordinated functional interactions between the endocrine system and the central and peripheral nervous systems, with commonalities at sub-cellular, cellular, and tissue levels. Such work allowed these systems to be incorporated into a universal diffuse neuroendocrine system (DNES) (Day and Salzet, 2002; Salzet and Day, 2003). The commonality in expression of biogenic amines and regulatory peptides may provide the basis for modeling of integrated functions across classically separated systems, given the location of DNES cells and their biologically active substances in most organs. DNES cells may play an important role in homeostatic regulation, with effects via neurocrine, endocrine, and paracrine mechanisms (Day and Salzet, 2002; Salzet and Day, 2003).

Other work showed that the nervous and immune systems have well-established interactions that regulate systemic homeostatic processes, driven by a number of cellular fluxes, including cytokines, chemokines, and peptides (El-Hattab and Scaglia, 2016; Turnbull and Rustin, 2016; Gutiérrez and Simmen, 2017). Such factors contribute to homeostatic regulation either via local effects or by the facilitation of non-local systems.

Most regulatory peptides and biogenic amines are present centrally, including in neurons and glia. Many immune cells, including macrophages, T-lymphocytes, eosinophilic leukocytes, and mast cells, can extravasate across the blood-brain barrier, especially when its barrier integrity has been compromised following brain injury or inflammation. Such immune cell extravasation is a significant contributor to central cytokines and other bioactive molecules, including some biogenic amines (Cruces et al., 2014; Souza et al., 2014; López-Griego et al., 2015; Gutiérrez and Simmen, 2017).

Such commonalities across the nervous, endocrine, and immune systems led to the field of neuroimmunoendocrinology, which primarily investigates the mutual inter-relationships between these regulatory systems (Peña-Blanco et al., 2017). Although the importance of neuroimmunoendocrinology is widely recognized, most investigations fail to collect data on all three aspects. Neuronal and immune cells as well as APUD cells are present in most visceral organs, where their products are similar to those released by these cells in the brain and organs of homeostatic regulation, including the thymus and thyroid gland.

It may then be possible to unite peptidergic/aminergic neurons-glia, APUD cells, and peptide-producing immunocompetent cells into a single common functional system, thereby extending DNES to a broader diffuse neuroimmunoendocrine system (DNIES). DNIES is especially important to the integration of signaling mechanisms underpinning homeostatic regulation. Alterations in homeostatic regulation are linked to almost all medical conditions, including classically defined neurodegenerative conditions, such as Alzheimer's disease (AD) and Parkinson's disease (PD) (Anderson and Maes, 2014a, 2017a).

## Classical pathophysiology of Alzheimer's and Parkinson's diseases

Neurodegenerative disorders, including AD and PD, show prominent mitochondrial dysfunction. The clinical importance of an improved understanding of the etiology and course of neurodegenerative disorders is highlighted by predictions estimating that over 20 million people are currently diagnosed with AD, a number that is expected to double by 2050 (Ziegler-Graham et al., 2008).

A triad of neuro-morpho-physiological features have classically been used to characterize AD, namely amyloid-β plaques (senile plaques), neurofibrillary tangles, and extensive neural loss, particularly in the hippocampus and cerebral cortex (Dickson, 1997). These changes correlate with dementia and the characteristic neurobehavioral changes arising in AD. The majority of AD cases arise sporadically, with a late life onset (after 65 years of age), although some show evidence of a familial early-onset dementia (40–50 years of age) (Ziegler-Graham et al., 2008).

PD is a not uncommon neurodegenerative disorder, with an end-point diagnosis based on the progressive loss of dopamine neurons in the pars compacta of the substantia nigra. Such dopamine neuron loss contributes to significant sensory and motor symptoms, including rigidity and tremor (Olanow and Tatton, 1999). Approximately 80% depletion of dopamine neurons in this region may occur before these symptoms become manifest, thought to arise from a PD course of at least 5–10 years (Olanow and Tatton, 1999). However, the etiology and course of PD may be longer given the proposed early etiology within the gut (Anderson et al., 2016b).

The classical pathophysiology of these common neurodegenerative conditions has emphasized end-point neuronal loss, with no clearly accepted perspective on the early etiology and course. Recent data indicate that wider body systems may have an important etiological role in neurodegenerative conditions, including a role for the gut and gut-brain axis (Anderson et al., 2016b).

## Molecular and cellular pathogenesis of neurodegenerative diseases

### Neurotrophic factors

Most neurotrophic factors belong to one of three families: (i) neurotrophins, such as brain-derived neurotrophic factor (BDNF) and nerve growth factor (NGF); (ii) glial cell-line derived neurotrophic factor family ligands; and (iii) neuropoietic cytokines, such as interleukin-6 (IL-6). They participate in the growth, differentiation, and survival of neurons, as well as in neurotransmission and plasticity (Gibon and Barker, 2017). Although normally having trophic effects, IL-6, via IL-6 trans-signaling, can also have pro-inflammatory effects (Anderson et al., 2013).

### Cytokines

Cytokine expression is rapidly up-regulated during tissue stress, contributing to both inflammatory processes and the restoration of tissue homeostasis. The most significant central source of pro-inflammatory cytokines, particularly after local damage, appears to be activated microglia, although neurons and astrocytes, as well as peri-vascular and endothelial cells can also release pro-inflammatory cytokines (Salvi et al., 2017). Cytokines modulate many central neurotransmitters, including noradrenaline, serotonin and GABA levels as well as the expression of a number of central neuropeptides, such as somatostatin, substance P, opioids, and vasoactive intestinal peptide (Neumann and Wekerle, 1998; Lori et al., 2017; Spitsin et al., 2017). However, the interrelationships among these varied neurotransmitter responses and their relevance to specific cytokine actions have still to be determined.

An important consequence of increases in the pro-inflammatory cytokines, IL-1β, IL-6, IL-18 and, especially interferon-γ, is their induction of indoleamine 2,3-dioxygenase (IDO). By driving tryptophan down the kynurenine pathway and away from the synthesis of serotonin and melatonin, IDO leads to a number of neuroregulatory products, including kynurenic acid (KYNA), and the excitotoxic quinolinic acid (QUIN). KYNA decreases excitatory glutamatergic activity, although may also inhibit the generally protective α7-nicotinic acetylcholine receptor (A7-nAChR), whilst other kynurenine pathway products generally have neurotoxic effects, as with QUIN (Anderson and Ojala, 2010). This is an important mechanism linking pro-inflammatory cytokine activity with alterations in neuronal activity and inter-area patterning, as well as in regulating mitochondrial function (Anderson and Maes, 2017b). The balance of the regulation of the kynurenine and serotonin/melatonin arms of tryptophan metabolism is a significant driver of alterations in neuroimmunoendocrinological functioning.

A number of second messenger systems in neurons and glia are also modulated by cytokines, as well as by melatonin and other hormones, including the protein kinases, nitric oxide (NO), arachidonic acid, and Ca2+ flux (Mrak et al., 1997; Kahya et al., 2017). As such, many of the key molecules linked to the pathogenesis of AD and other mitochondrial diseases may be modulated by cytokines and wider regulatory peptides.

**Figure 1:**
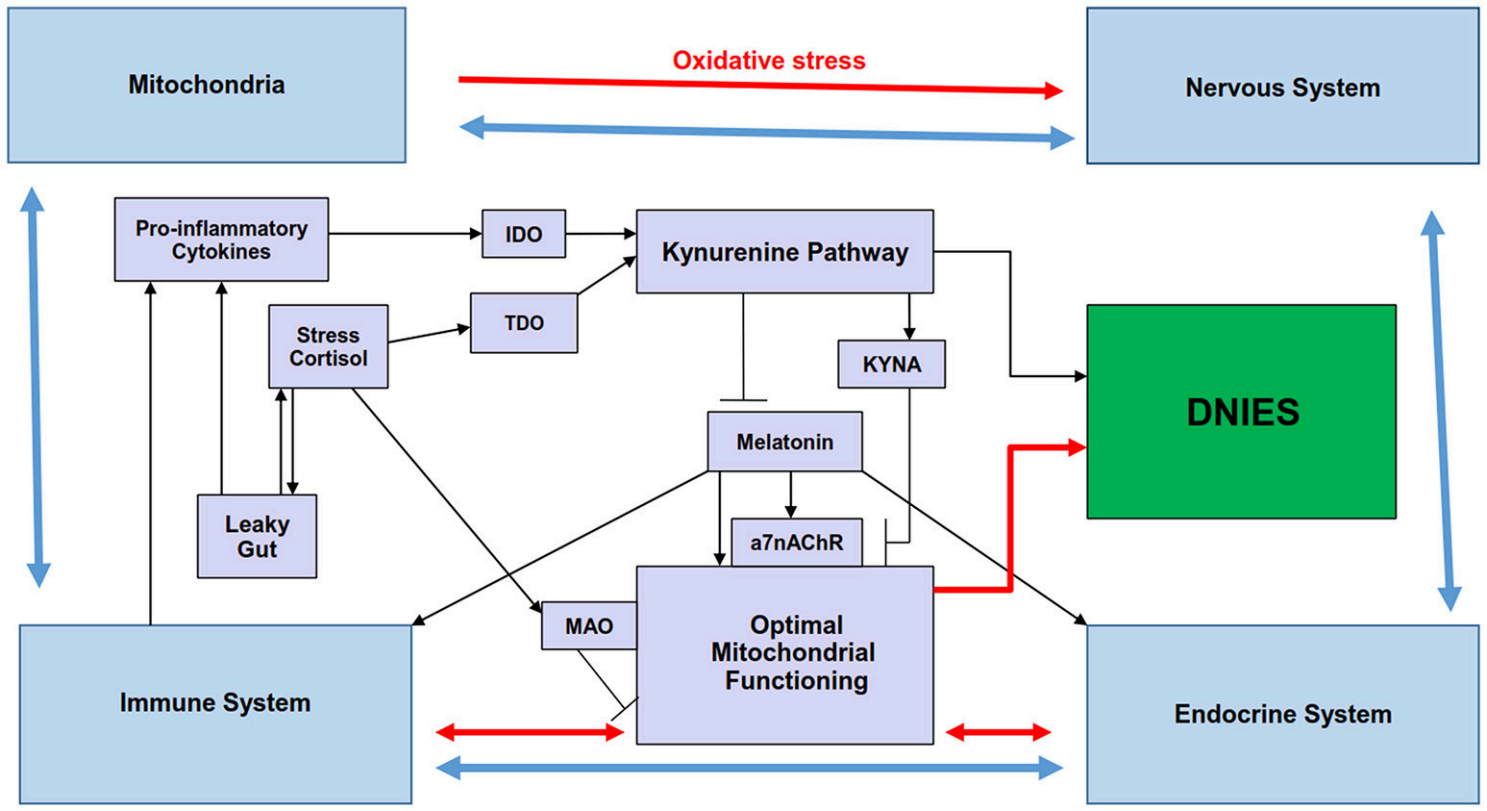
Summary scheme showing how the regulation of melatonin and the kynurenine pathways by stress and inflammation may be involved in the reciprocated interactions of mitochondria and the immune, endocrine, and nervous systems that comprise the diffuse neuroimmunoendocrine system (DNIES). Pro-inflammatory cytokines and chronic stress increase IDO and TDO, respectively, leading to kynurenine pathway induction, with differential effects on mitochondrial functioning (black lines). Kynurenine pathway activation and stress-induced MAO, by decreasing serotonin availability, decrease melatonergic pathway activation, thereby contributing to suboptimal mitochondrial functioning. KYNA inhibits the a7nAChR, thereby inhibiting some of melatonin's effects, which may be mediated via the a7nAChR (black lines). The interactions of the endocrine, immune and nervous systems (blue lines) are modulated by mitochondrial functioning (red lines), with the effects of these systems and their interactions differentially feeding back on mitochondrial functioning in different cells of these systems. Such reciprocated interactions form the DNIES. The kynurenine pathway is also intimately connected to the component systems of DNIES. a7nAChR, alpha 7 nicotinic receptor; IDO, indoleamine 2,3-dioxygenase; MAO, monoamine oxidase; TDO, tryptophan 2,3-dioxygenase.

As indicated above many cytokines can exert neurotrophic, neuroprotective, and neurotoxic actions, as exemplified by IL-6. Transgenic mice over-expressing IL-6 in astrocytes show marked neurodegeneration, which is partially prevented by the inhibition of IL-6, as well as IL-1β inhibition. The inflammasome linked induction of IL-1β increases the β cleavage of amyloid precursor protein (β-APP) and adhesion molecules in neural tissue (Yirmiya and Goshen, 2011; Meraz-Ríos et al., 2013; Singhal et al., 2014). As noted previously, increased IL-6 as well as the other inflammasome products, IL-1β and IL-18, will concurrently increase IDO levels, thereby activating the kynurenine pathway products, whilst concurrently decreasing levels of serotonin and melatonin (Anderson et al., 2016a) (Figure 1). The activation of such processes has significant consequences for mitochondrial functioning (Anderson and Maes, 2017b).

Many hormones influence cytokine actions, including: glucocorticoids, which are potent inhibitors of cytokine synthesis and effects; melanocyte stimulating hormone and vasopressin, which attenuate central actions of cytokines; and melatonin, which acts to decrease the synthesis and effects of pro-inflammatory cytokines. These, and other, peptides have a role in the inappropriate febrile response that is initiated by cytokines in older animals (Brough et al., 2015; Pradillo et al., 2017). As such, there are a wide range of communications across different cells, tissues, and organs that have implications for mitochondrial functioning, including within these cells and their higher order systems. Alterations in the interactions of mitochondria functioning with DNIES systems contributes to the pathophysiology of AD and PD.

The classical pathophysiological changes in AD are an increased expression of senile plaques and neurofibrillary tangles (Dickson, 1997). However, it should be noted that a number of controversies exist as to the nature of AD and its pathology, including: is AD a heterogenous collection of different disorders with a common cognitive presentation? is amyloid-β synthesis a failed attempt at neuro-protection? is there a primary increase in neurofibrillary tangles, followed sequentially by amyloid-β production? do changes initially occur out with the brain, e.g., in the gut, driven by stress and depression (Anderson and Maes, 2017a) or in the retina and circadian regulation (La Morgia et al., 2017). Such questions highlight the complexity in the conceptualizations of AD, including complexity arising from interactions across different organs, tissues, and biological systems.

### Amyloid β (Aβ)

Although tempered by such wider questions, the relationship between amyloid deposits and neurofibrillary tangles, as well as their interactions with wider inflammatory processes, are widely recognized as important areas of investigation in AD pathogenesis (Selkoe, 1998; Dodd et al., 2013; Zhao et al., 2017).

AP can be readily reproduced in transgenic mice. These mice exhibit selective neuronal death in the brain regions that are most affected in AD, implicating AP formation in AD neuron loss (Rupp et al., 2011; Schafer et al., 2015). It is also important to note that reactive astrocytes produce Aβ, including when toll-like receptor-4 is activated (Gong et al., 2014). As such, systemic processes that increase blood-brain barrier permeability and/or increase astrocyte reactivity will contribute to Aβ and neuronal apoptotic susceptibility, including alterations in the gut microbiome and increased gut permeability (Rothhammer et al., 2016). Aβ is increased in a number of other conditions, with a non-Aβ component of human α-synuclein oligomers inducing the formation of new Aβ oligomers, indicating overlaps in the mechanisms classically associated with AD and PD (Atsmon-Raz and Miller, 2016).

### Tau-protein

Data indicates that even low levels of neurofibrillary tangles are pathological and may be an early contributor to the etiology of AD. Such filamentous lesions are often evident in other neurodegenerative diseases, including PD, Down syndrome, and myotonic dystrophy.

Within nerve cells tau protein is mainly present in axons (Scholz and Mandelkow, 2014; Zetterberg, 2017) although tau pathology is also present in the fibroblasts of AD patients (Mukhamedyarov et al., 2016), as well as in other medical conditions (Takeda, 2017). It is of note that tau filaments are commonly released by neurons, being picked up by astrocytes, which drives changes in gliotransmitter release, in turn altering neuronal activity and survival (Piacentini et al., 2017). It is also important to note that tau pathology leads to suboptimal mitochondrial functioning (Pérez et al., 2017), suggesing that tau pathology may contribute to wider system changes and interactions, in part by alterations in mitochondrial functioning.

### Synuclein proteins

Intracellular protein accumulations, or inclusions, are common across an array of neurodegenerative conditions, including Lewy bodies (LB) in PD. LB are α-synuclein-positive in PD patients (Spillantini et al., 1997). In addition to synuclein, LB contain ubiquitin, ubiquitin C-terminal hydrolase, and proteasomal subunits, which are major components of the cellular protein degradation pathway (Alves-Rodrigues et al., 1998; Pallares-Trujillo et al., 1998). In PD, α-synuclein levels are highest in the putamen, substantia nigra, locus coeruleus, inferior olive, pons, and cerebellum.

The brain distribution of α-synuclein significantly overlaps with wider AD brain pathology (Atsmon-Raz and Miller, 2016). Additional portions of the synuclein protein are present in amyloid plaques in AD. Enhanced levels of cerebral spinal fluid α-synuclein are evident in AD, futher implicating a-synuclein in the development of AD, as well as its classical association with PD and LB dementia (Chiasserini et al., 2017).

Recent data, suggesting a more whole body perspective of PD, proposes that α-synuclein may be formed in the gut and transported to the brain, possibly involving the vagal nerve, whilst alterations in substanti nigra pars compacta functioning can also modulate gut functioning (Anderson et al., 2016b; Anselmi et al., 2017; Chandra et al., 2017; Choudhry and Perlmuter, 2017). Enteroendocrine cells (EECs) are part of the gut lumen-facing epithelium, with EECs possessing many neuron-like properties and connections to enteric neurons. α-synuclein is expressed in murine and human EECs that directly connect to α-synuclein-containing neurons (Chandra et al., 2017). This suggests the formation of a neuronal circuit between the gut and the nervous system, whereby toxins or other environmentally influenced factors in the gut lumen can modulate α-synuclein folding in the EECs. Such a model implicates the gut as a potential primary site in the etiology of PD (Anderson et al., 2016b), thereby beginning a process by which misfolded α-synuclein could propagate from the gut epithelium to the brain. Recent data also shows that gut α-synuclein increases inflammatory processes in the gut, which are also proposed to contribute to the pro-inflammatory milieu in PD (Stolzenberg et al., 2017). In this context, it is important to note that tau pathology can lead to suboptimal mitochondrial functioning (Pérez et al., 2017), suggesting a spreading suboptimal mitochondrial functioning following tau pathology from gut to brain.

Such previously unrecognized reciprocal interactions between such distinct areas, substantia nigra and gut, indicate significant alterations in conceptualizations of neurodegenerative conditions. It is also important to note that the changes occuring in mitochondria are likely to have impacts on other systems and inter-area interactions, thereby altering the homeostatic regulation occurring in DNIES.

## Integrating central and systemic processes in neurodegenerative conditions

As indicated above, the classical modeling of neurodegenerative conditions has focused on central changes, including in regard to suboptimal mitochondrial function. Recent work, indicating the value of a more wholistic perspective, suggests that the etiology of conditions such as AD and PD may be, at least in part, in peripheral organs and tissues, such as the gut and retina (Anderson et al., 2016b; La Morgia et al., 2017). The processes by which such peripheral changes drive alterations in the brain is the subject of intense investigations, including: the inter-organ transfer of inclusions, such as α-synuclein transport along neurons; the induction of local systemic inflammatory processes, leading to an increase in circulating pro-inflammatory cytokines that can regulate central processes; increased pro-inflammatory cytokines induction of IDO, leading to an increase in systemic kynurenine that can be transferred over the blood-brain barrier, in turn driving the formation of neuroregulatory kynurenine pathway products (Maes and Anderson, 2016); impacts via the regulation of the vagal nerve (Ulusoy et al., 2017); and pathways involving alterations in diffuse endocrine cell activity and functioning.

It is clear that alterations in immune system functioning may be integral to the classical changes linked to CNS conditions, such as AD and PD. Neuronal activity may be conceptualized as a form of immune-to-immune communication, including glia as central immune cells (Anderson, 2011), with interactions of both immune-glia and neurons with endocrine processes (Anderson et al., 2012). It is clear that defining CNS disorders by changes occurring in the brain, occludes the role of systemic processes in the developmental etiology of these classically defined brain conditions. Consequently, it is likely that many of the genetic susceptibilities to AD and PD are mediated via their influence on such systemic processes, and not necessarily in the brain, as has been classically modeled. Such modeling also allows for epigenetic and environmental impacts to be mediated via changes in systemic sites and processes. It may be useful to learn from this history. For example, the rush to link retina and gut changes directly to alterations in the brain may also be premature, perhaps involving peripheral organ-to-organ interactions, before central consequences arise, such as interactions of the oral cavity with the gut (Gomes et al., 2017). It is in this wider interactive ‘wholistic’ perspective that DNIES must be placed.

It is clear that alterations in mitochondrial function occur in almost all CNS conditions, including AD and PD. As indicated above, most of this work has focused on suboptimal mitochondrial function in different brain regions, such as in the cortex and substantia nigra in AD and PD, respectively. However, it is important to note that recent work shows mitochondria to have a crucial role in immune cell functioning (Mehta et al., 2017). Evolutionary pressures have provided an over-riding influence of the immune system, given its crucial role in survival. As such, alterations in immune cell functioning will have significant impacts on cellular, tissue, and organ processes. Most medical conditions have some aspect of key symptomatology that is driven by changes in the immune system, including cancers, as well as cardiovascular, arthritic, and CNS disorders. Alterations in immune cell mitochondrial function are therefore likely to have significant implications across a host of medical conditions (Anderson and Maes, 2017b). Of note, mitochondria have been proposed as an important hub for the direct interactions of a wide array of factors linked to an array of medical conditions, including psychiatric conditions (Anderson, 2017).

By driving down the levels of serotonin and melatonin, the pro-inflammatory cytokine induction of IDO may have significant impacts on mitochondrial function. A plethora of data shows melatonin to optimize the functioning of mitochondria, in part by increasing levels of sirtuins, which are important mitochondrial regulators (Guo et al., 2014), with mitochondria-located sirtuin-3 rescuing dopamine neurons in an α-synuclein model of PD (Gleave et al., 2017). Importantly, melatonin may also be produced within mitochondria (He et al., 2016), suggesting that DNIES processes that drive increases in kynurenine pathway activity, may also act to regulate the levels of melatonin within mitochondria, and therefore the optimization of mitochondrial function. This will be important to clarify, including as to the relevance of melatonin accumulation on the protruding lipid arms of the outer mitochondrial membrane (García et al., 2011), including in regard to the complexes formed on the outer membrane, which determine the initiation of apoptosis. As such, DNIES may be making important contributions to an array of medical conditions by direct effects in mitochondria, including from related changes in the levels of melatonin and kynurenine pathway products (Anderson, 2017). The relevance of this to the functioning of different immune cells will be important to determine, given data showing that it is the autocrine effects of melatonin that shifts macrophages from a pro-inflammatory M1-like phenotype to an anti-inflammatory, pro-phagocytic M2-like phenotype (Muxel et al., 2012).

It is also important to note that this is a reciprocated relationship, with changes in mitochondrial activity and metabolic products acting to regulate the levels of inflammasome-driven pro-inflammatory cytokines, especially IL-1β and IL-18, which can increase IDO and drive alterations in immune functioning and neuroregulatory kynurenine pathway products (Anderson and Rodriguez, 2011; Próchnicki and Latz, 2017). Mitochondria may therefore be an important hub for the neuroimmunoendocrine interactions occurring in DNIES, thereby intimately linking alterations in mitochondrial function with plethora of data and processes linked to neuronal, immune and endocrine investigations.

The formation of dopamine is associated with an increase in oxidant production, which contributes to the apoptotic susceptibility of substantia nigra dopamine neurons in PD (Herrera et al., 2017). Recent data shows that dopamine is also an important regulator of the immune system, including by its release from T cells that regulate antibody production in interacting B cells (Papa et al., 2017). This involves a series of interactions that require T cell dopamine release to optimize B cell antibody production. Another biogenic amine, norepinephrine (NE), is also an important regulator of immune cell functioning, via its effects on mitochondrial superoxide production (Case et al., 2016). In purified murine splenic CD4+ and CD8+ T-lymphocytes that were NE-stimulated, the levels of mitochondrial superoxide production determined the levels of cytokines produced (Case et al., 2016). Such data highlights the importance of mitochondrial metabolism in the regulation of immune responses, including in cytokine-induced IDO and kynurenine pathway activity that are linked to a plethora of diverse medical conditions (Morris et al., 2016).

Another biogenic amine, serotonin, also regulates mitochondrial function via the modulation of superoxide production, which requires upstream 5-HT2 receptor activation (Genet et al., 2017). As such, serotonin has effects on mitochondrial function, directly via its 5-HT2 receptor as well as by acting as a necessary precursor for melatonin synthesis. Another biogenic amine, histamine, modulates the important Ca2+ transfer between the endoplasmic reticulum and mitochondria (Gomez et al., 2016). Such Ca2+ transfer is of some importance to mitochondria and wider cellular functioning. Overall, such data suggests that the biogenic amines, evident in neuronal, immune and endocrine cells, are important modulators of mitochondrial function, with consequences not only for cell functioning, but for the functioning of other neuronal, glia/immune, and endocrine cells. As such, DNIES is intimately linked to mitochondrial function and, consequently, with the wide array of mitochondrial disorders, including AD and PD.

Overall, DNIES may be intimately related to the integration of the signaling mechanisms that underpin homeostatic regulation by the widespread interaction of these biogenic amines and peptides with local mitochondrial function, which in turn, determines the functioning of the diverse array of cells that make up the DNIES. DNIES and mitochondrial function may therefore be closely related. This has implications for a host of different factors that have been linked to many medical conditions, including chemokines, integrins, Ca2+ regulation, and apoptotic processes.

### Chemokines

Chemokines and chemokine receptors have low constitutive expression in astrocytes, microglia and neurons of the developing and adult brain, being up-regulated by inflammatory mediators (Cacabelos et al., 2016), including in AD and other CNS diseases (Cacabelos et al., 2016). Along with other inflammatory factors, chemokines may be intimately linked to variations in mitochondrial function (Monlun et al., 2017).

### Integrins

Integrins mediate attachment to the extracellular matrix and cell-cell adhesion. By virtue of their barrier functions, changes in integrin levels, subtypes, and function are closely linked to alterations in inter-area communications. This links integrins to many key processes and medical conditions (Banerjee et al., 2017; Niu and Li, 2017). Of note, integrins may be intimately linked to mitochondrial function (Visavadiya et al., 2016), with alterations in mitochondrial function linked to changes in integrins (Nunes et al., 2015).

### Exosomes

Exosomes are now recognized as being released from a growing number of different cell types, with their variable content thought to represent alterations in cellular functioning. The inclusion of microRNA in exosomes allows one cell to influence the transcription of other cells and cell types, including in the regulation of integrins and blood-brain barrier permeability (Zhao and Zlokovic, 2017). Alterations in the content of exosomes can regulate memory dysfunction in AD models (Dinkins et al., 2016), whilst changes in mitochondrial function are linked to alterations in exosome content (Eitan et al., 2016).

### Chaperones

Investigations of mitochondrial protein biogenesis have shown chaperones to act as unfoldases, foldases and pulling devices. Endoplasmic reticulum (ER) chaperones and folding assistants, via ER-mitochondria Ca2+-flux, act to regulate mitochondrial metabolism, and thereby cellular apoptosis (Gutiérrez and Simmen, 2017). Molecular chaperones, including the heat shock proteins, act to regulate key processes in AD (Lackie et al., 2017), with mitochondrial import stimulation factor (Marada et al., 2013) being an important conformational modulator of mitochondrial precursor proteins. Other heat shock proteins (Ssa1p, Ssa2p, and especially Hsp70) contribute to protein import into mitochondria, ER and nuclei (Veereshwarayya et al., 2006; Marada et al., 2013). Hsp70 is also an important regulator of the immune inflammatory response (Khandia et al., 2017). The data on hps70 and other chaperones highlights the role of mitochondrial regulators in wider system changes.

A number of other factors that directly interact with mitochondria and/or mitochondrial function can significantly modulate DNIES and include: neurosteroids, which are synthesized following the mitochondrial uptake of cholesterol (Selvaraj and Tu, 2016; Bak, 2017; De Nicola et al., 2017; Solanki et al., 2017; Tuem and Atey, 2017); ROS-driven DNA damage leading to changes in mitochondrial metabolism (Brace et al., 2016), with mitochondrial DNA damage linked to aging and increased apoptotic susceptibility (Mani et al., 2017; Sanabria-Castro et al., 2017); iNOS-derived NO mediating the mitochondrial and dopamine neuron damage by α-synuclein in PD models (Tapias et al., 2017), with NO acting as a relevant regulator of neuronal, immune-glia, and endocrine systems (Costa et al., 2016); glutamate and kynurenine pathway products activation, leading to excessive excitatory transmission, arising from resultant increased mitochondrial Ca2+-influx and alterations in ER-mitochondria Ca2+ regulation (Rueda et al., 2016).

### Apoptosis

The process of programmed cell death, or apoptosis, underlies most cell death, including neuronal death in neurodegenerative disorders, such as AD and PD. Apoptosis is tightly linked to mitochondrial processes, through the effects on mitochondrial function of pro- and anti-apoptotic small proteins, such Bax and Bcl-2, respectively. The formation of the mitochondrial permeability transition pore involves the formation of a complex on mitochondria leading to the release of cytochrome c and caspase activation. As such, alterations in mitochondrial function may be intimately linked to the threshold for driving cell death. This links to recent conceptualizations of apoptosis, where the spatial temporal interactions of a number of factors on the mitochondrial outer membrane can determine apoptotic susceptibility (Cosentino and García-Sáez, 2017).

Overall, it is clear that mitochondrial function is crucial to the survival and activity of the neuronal, glia-immune and endocrine cells that make up the DNIES. In turn, alterations in the functioning of these interacting DNIES components can have significant impacts on mitochondrial function in different cell types. An array of different factors underlies the interactions of the constituent effectors of DNIES, such as exosomes, cytokines, kynurenine pathway products, biogenic amines, and chemokines, with important impacts mediated in mitochondria, including levels of ROS and oxidative phosporylation as well as ER-mitochondria Ca2+ regulatory interactions. Such data makes it clear why mitochondrial disorders, as widely defined, includes such a wide array of distinct medical conditions.

## Central and systemic hormones: roles in DNIES and mitochondrial diseases

As indicated above, an increase in pro-inflammatory cytokines drives tryptophan to the production of neuroregulatory kynurenine pathway products and away from serotonin synthesis. As serotonin is also the precursor of N-acetylserotonin (NAS) and melatonin, there will be a coordinated decrease in the levels of NAS and melatonin following such inflammation-driven decrease in serotonin synthesis.

### Melatonin

Melatonin is classically associated with the regulation of circadian rhythms following its night-time release by the pineal gland (Pfeffer et al., 2017). However, a growing body of data shows melatonin to be released by a plethora of different cell types, including astrocytes, mast cells, natural killer cells, eosinophilic leukocytes, platelets, epithelial cells, endothelial cells, and bone marrow cells (Kvetnoy, 1999; Mazzoccoli et al., 2010, 2011; Anderson and Maes, 2014b; Paltsev et al., 2016). Recent work indicates that melatonin may be produced by all mitochondria-containing cells (Tan et al., 2013), and perhaps within mitochondria (Mehta et al., 2017). This is of some importance as melatonin, as well as being an antioxidant, anti-inflammatory, and anti-nociceptive, is also an optimizer of mitochondrial function (Anderson, 2017). Melatonin is amphiphilic, thereby able to transverse through the extracellular matrix and within cells, as well as across cell membranes. Melatonin also has effects via melatonin receptors. Such data indicates that variations in melatonin synthesis will have antioxidant and anti-inflammatory consequences, including via autocrine and paracrine effects, with significant impacts on mitochondrial function across different systems. Some of the impacts of increased pro-inflammatory cytokines across a host of medical conditions, will be mediated by a decrease in the mitochondria-optimizing effects of melatonin.

Melatonin has utility in the treatment of a host of medical disorders, including AD, PD, cancers, endometriosis, multiple sclerosis, and psychosis (Anderson and Maes, 2012, 2014c, 2015a; Maes and Anderson, 2016; Rodriguez et al., 2016; Terraneo et al., 2017). Such data has arisen from the utility of melatonin when given at night, usually targeted to improve sleep across different disorders. Given that melatonin can decrease the reactivity of immune cells (Muxel et al., 2012), the targeting of local melatonin synthesis in different cell types is likely to significantly modulate the mitochondrial dysfunction evident in DNIES systems across different medical conditions. The amphiphilic nature of melatonin makes it an ideal candidate for communications across neuronal, glia-immune and endocrine systems, with its levels being modulated by mitochondrial function as well as modulating mitochondrial function across the systems forming DNIES. As to whether the structural changes in mitochondria mediated by melatonin are relevant to how complexes gather on the mitochondrial outer membrane in the modulation of apoptosis requires further investigation.

The classical role of pineal melatonin night-time release may also be important to DNIES, especially regarding immune-inflammatory processes. An increase in pro-inflammatory cytokines can dramatically decrease pineal melatonin release (Pontes et al., 2007). This has led to the conceptualization of an immune-pineal axis, whereby ongoing, and necessary, pro-inflammatory activity prevents the release of melatonin and its anti-inflammatory effects.

Melatonin exerts multiple actions in the modulation of the immune system function impacting the innate and the acquired response and in turn is produced by immunocompetent cells (Carrillo-Vico et al., 2005; Calvo et al., 2013).

Pineal melatonin decreases the rolling and adhesion of immune cells on endothelial cells, and therefore would lower necessary immune cell chemo-attraction to sites where required. As such, the circadian regulation by melatonin is intimately linked to variations in ongoing infections and tissue damage.

### N-acetylserotonin (NAS)

NAS has similar effects to melatonin, including antioxidant and anti-inflammatory, as well as being amphiphilic and positively regulating mitochondrial function. Although NAS is usually released at similar concentrations to melatonin, it has been far less studied. NAS is a BDNF mimic, through its activation of the BDNF receptor, TrkB (Jang et al., 2010). However, in some medical conditions, variations in the NAS/melatonin ratio may be of some significance. For example, in glioblastoma, bipolar disorder, and endometriosis, an increase in TrkB activation is detrimental, suggesting that alterations in the NAS/melatonin ratio will be of some importance in these conditions (Anderson and Maes, 2015a,b; Beischlag et al., 2016).

### Serotonin

Although serotonin has many effects via its many receptors, it is important to note that the regulation of melatonin, either by its uptake by serotonin transporters or its breakdown by monoamine oxidase (MAO), will have significant impacts on the levels of NAS and melatonin synthesis. Many of the factors known to have beneficial effects across an array of medical conditions, such as caffeine, taurine, green tea's epigallocatechin gallate, and curcumin, decrease MAO levels, thereby increasing serotonin availability, including for NAS and melatonin synthesis (Calhoun et al., 2017). The importance of this issue to the beneficial impacts of such nutraceuticals is the subject of ongoing investigation. Likewise many pharmaceuticals with relatively wide-ranging benefits, such as antidepressants, may be mediating their effects on levels of NAS and melatonin availability. It is clear that such nutraceutical and pharmaceutical regulation of NAS and melatonin, as well as serotonin and other monoamines, will have direct impacts on neuronal, glia-immune and endocrine cell functioning, as well as on their interactions, at least in part mediated by mitochondrial regulation. It is also of note the MAO is expressed on the mitochondrial outer membrane.

Decreased serotonin levels have classically been associated with depression. Many medical conditions are associated with prodromal depression, including AD and PD, as well as exacerbations in relapse-remitting multiple sclerosis (Anderson and Maes, 2017a). The significant decrease in serotonin neurons in the AD cortex (Stefano et al., 2016), leads not only to a loss of serotonin's neuromodulatory effects, but also to a decrease in the levels of serotonin as a precursor for NAS and melatonin, thereby modulating mitochondrial function.

### Other proteins

Other neuromodulatory transmitters, including dopamine, NE and histamine show alterations in neurodegenerative conditions, such as AD and PD. All are associated with mitochondrial function (Vuda et al., 2012; Luo et al., 2013). Although histaminergic neurons are present in the brain, the majority of research has investigated mast cell release of histamine, as well as melatonin. Mast cells may be significant cells in DNIES and mitochondrial diseases, given that they are frequently found at important barriers, including the gut barrier (Vanuytsel et al., 2014) and blood-brain barrier (Zhang et al., 2016), both of which they can significantly modulate. Mast cell function and degranulation is significantly modulated by mitochondria (Cuong et al., 2016), whilst mast cells can also release mitochondrial components into the general circulation, with potential autocrine and paracrine inflammatory consequences (Zhang et al., 2012).

A number of other peptides show alterations in medical conditions characterized by suboptimal mitochondrial function, including: somatostatin, which may inhibit the hyperglycemic response to a variety of stressors and is co-expressed with low-density lipoprotein receptor-related protein (LRRP) in hippocampal and cortex interneurons; endogenous opioids, with the human δ-opioid receptor modulating both *A*β production (Sarajärvi et al., 2015) and mitochondrial function (Zhu et al., 2009); corticotrophin-releasing hormone, adrenocorticotropin hormone and corticosteroids, which can significantly modulate mitochondrial function, including in the process of T-cell apoptosis (Prenek et al., 2017). Acute vs. chronic glucocorticoids differentially regulate MAO, linking to mitochondrial differences in acute vs. chronic stress (Morris et al., 2017).

Alterations in steroid hormones are linked to a plethora of medical conditions, with steroid hormone synthesis being dependent upon cholesterol uptake into mitochondria, involving the steroidogenic acute regulatory protein (StAR) on the outer mitochondrial membrane. StAR activity requires a multi-component complex on the outer mitochondrial membrane, including the 18 kDa translocator protein and the voltage-dependent anion channel, the latter being an important component of the mitochondrial permeability transition pore. This complex is involved in the transport of cholesterol for steroid hormone synthesis by cytochrome P450 cholesterol side-chain cleavage enzyme (Miller, 2013). Mitochondrial dysfunction is associated with alterations in steroid hormone synthesis, including in macrophages, with relevance for levels of immune system activity (Graham, 2015).

Overall, alterations in mitochondrial functioning are intimately linked in a reciprocated relationship with DNIES. Given that mitochondria in the systems comprising DNIES are important drivers of system functioning, such interactions may be viewed as a form of mitochondria-to-mitochondria communication. Alterations in mitochondria function at different sites and in different systems will therefore have significant impacts on homeostatic regulation. This may be parsimonius with current evolutionary conceptualizations, whereby bacteria gradually become modified to form mitochondria in the first eukaryotic cell. Evolutionary forces have therefore acted on the transition from inter-bacteria to inter-mitochondria communication, which would seem to form the basis of DNIES, homeostatic regulation and the dysregulation occuring in AD and PD, as well as in many other medical conditions.

## Conclusions

In summary, it is clear that the neuronal, glia-immune and endocrine systems that form DNIES are intimately and reciprocally linked to mitochondrial function, with consequences for the etiology, course and management of a growing list of medical conditions associated with mitochondrial dysfunction. This may be of particular importance at sites that may be seen as crucial hubs, such as the gut, BBB and vagal nerve, as well as mitochondria *per se*. Melatonin impacts at multiple levels on the interactions of DNIES and mitochondrial function, suggesting that local melatonin synthesis targeted to particular cells and sites may be an important pharmaceutical target. Such a frame of reference provides a more wholistic perspective on medical conditions that have classically been understood and treated within a particular organ or tissue, such as the brain in AD and PD.

## Author contributions

All authors listed have made a substantial, direct and intellectual contribution to the work, and approved it for publication.

### Conflict of interest statement

The authors declare that the research was conducted in the absence of any commercial or financial relationships that could be construed as a potential conflict of interest.
